# Supplementary material for: Mapping the evolution of fertility support policies in China: A content and instrumental analysis
Source: PLoS One. 2025 Oct 9;20(10):e0332137. doi: 10.1371/journal.pone.0332137 (PMC12510515; doi:10.1371/journal.pone.0332137)
Supplement: S1 Appendix — (ZIP) [file pone.0332137.s001.zip › S1 Appendix. 226 original policy documents/112-人力资源社会保障部、财政部、国家卫生计生委关于做好当前生育保险工作的意见(FBM-CLI-4-311345).doc]

人力资源社会保障部、财政部、国家卫生计生委关于做好当前生育保险工作的意见

**发布部门：** [人力资源和社会保障部](https://www.pkulaw.com/law/chl?Aggs.IssueDepartment=60236) [财政部](https://www.pkulaw.com/law/chl?Aggs.IssueDepartment=60210) [国家卫生和计划生育委员会(已撤销)](https://www.pkulaw.com/law/chl?Aggs.IssueDepartment=60319) [机构沿革](javascript:void(0))

**发文字号：**人社部发〔2018〕15号

**发布日期：**2018.03.05

**实施日期：**2018.03.05

**时效性：** [现行有效](https://www.pkulaw.com/law/chl?Aggs.TimelinessDic=01)

**效力级别：** [部门规范性文件](https://www.pkulaw.com/law/chl?Aggs.EffectivenessDic=XE0302)

**法规类别：** [人身保险](https://www.pkulaw.com/law/chl?Aggs.Category=0440301)

人力资源社会保障部、财政部、国家卫生计生委关于做好当前生育保险工作的意见
（人社部发〔2018〕15号）

各省、自治区、直辖市及新疆生产建设兵团人力资源社会保障厅（局）、财政厅（局）、卫生计生委，福建省医保办：
　　生育保险制度自建立以来，总体保持平稳运行，对维护职工生育保障权益、促进妇女公平就业、均衡用人单位负担发挥了重要作用。近年来，为应对经济下行压力，生育保险采取降费率措施，减轻了企业负担；同时，应对人口老龄化，适应国家实施全面两孩政策，采取措施保障生育保险待遇，促进了人口均衡发展。当前，为切实维护全面两孩政策下参保职工合法权益，确保生育保险稳健运行，现对进一步做好生育保险工作提出如下意见：
　　**一、**提高认识，确保生育保险待遇落实
　　实施全面两孩政策是适应人口和经济社会发展新形势的重大战略举措，落实生育保险政策是实施全面两孩政策的重要保障措施。各地要统一思想，提高认识，主动适应计划生育政策调整，坚持科学发展，体现社会公平，切实维护职工合法权益。要确保应保尽保，将符合条件的用人单位及职工纳入参保范围；确保参保职工的生育医疗费用和生育津贴按规定及时足额支付，杜绝拖欠和支付不足现象。要根据全面两孩生育政策对生育保险基金的影响，增强风险防范意识和制度保障能力，确保生育保险基金收支平衡，实现制度可持续发展。

　　**二、**加强预警，完善费率调整机制
　　各地要结合全面两孩政策实施，完善生育保险监测指标。充分利用医疗保险信息网络系统，加强生育保险基金运行分析，参照基本医疗保险基金管理要求，全面建立生育保险基金风险预警机制，将基金累计结存控制在6-9个月支付额度的合理水平。
　　基金当期入不敷出的统筹地区，首先动用累计结存，同时制定预案，根据《[社会保险基金财务制度](https://www.pkulaw.com/chl/e4e06e49beb752e2bdfb.html)》提出分类应对措施，经报同级政府同意后及时启动。基金累计结存不足（﹤3 个月支付额度）的统筹地区，要及时调整费率，具体费率由统筹地区按照“以支定收、收支平衡”的原则，科学测算全面两孩政策下基金支出规模后合理确定。基金累计结存完全消化的统筹地区，按规定向同级财政部门申请补贴，保障基金当期支付，同时采取费率调整措施，弥补基金缺口。
　　开展生育保险与职工基本医疗保险（以下统称两项保险）合并实施试点的统筹地区，要通过整合两项保险基金和统一征缴，增强基金统筹共济能力。要跟踪分析合并实施后基金运行情况，根据基金支出需求，确定新的费率并建立动态调整机制，防范风险转嫁。

　　**三、**引导预期，规范生育津贴支付政策
　　各地要按照“尽力而为、量力而行”的原则，坚持从实际出发，从保障基本权益做起，合理引导预期。要综合考虑生育保险基金运行和用人单位缴费等情况，规范生育津贴支付期限和计发标准等政策，确保基金可持续运行和待遇享受相对公平。确保《[女职工劳动保护特别规定](https://www.pkulaw.com/chl/cdf82292dbb62ee9bdfb.html)》法定产假期限内的生育津贴支付，探索多渠道解决生育奖励假待遇问题。

　　**四、**加强管理，提高基金使用效率
　　各地要结合全民参保计划实施，进一步扩大生育保险覆盖面，加大征缴力度，与基本医疗保险同步推进统筹层次提升。加强生育保险定点协议管理，切实保障参保人员生育医疗权益，促进生育医疗服务行为规范。将生育医疗费用纳入医保支付方式改革范围，实行住院分娩医疗费用按病种、产前检查按人头付费，实现经办机构与定点医疗机构费用直接结算。充分利用医保智能监控系统，强化监控和审核，控制生育医疗费用不合理增长。

　　**五、**高度重视，切实做好组织实施工作
　　各地要高度重视生育保险工作，切实加强组织领导，做好统筹协调。加强政策宣传与舆论引导，准确解读相关政策，及时回应群众关切。各级人力资源社会保障、财政、卫生计生部门要明确职责，密切配合，形成工作合力，加强对统筹地区工作指导，及时研究解决有关问题。积极稳妥推进两项保险合并实施试点工作，及时总结试点经验，为全面推开两项保险合并实施工作奠定基础。工作推进中，如遇到重大问题，要及时报告。

人力资源社会保障部
财政部
国家卫生计生委
2018年3月5日

© 北大法宝：（[www.pkulaw.com](https://www.pkulaw.com/)) 专业提供法律信息、法学知识和法律软件领域各类解决方案。北大法宝为您提供丰富的参考资料，正式引用法规条文时请与标准文本核对。
欢迎查看所有[产品和服务](https://www.pkulaw.com/helps/2.html)。法宝快讯：[如何快速找到您需要的检索结果？](https://www.pkulaw.com/helps/70.html)[法宝](https://www.pkulaw.com/helps/69.html)V6有何新特色？

扫描二维码阅读原文

**原文链接：**<https://www.pkulaw.com/chl/29db538d92c04310bdfb.html>
